# The Role of Cytokines in Predicting the Response and Adverse Events Related to Immune Checkpoint Inhibitors

**DOI:** 10.3389/fimmu.2021.670391

**Published:** 2021-07-22

**Authors:** Min Wang, Xiaoyang Zhai, Ji Li, Jingyuan Guan, Shuhui Xu, YuYing Li, Hui Zhu

**Affiliations:** ^1^ Department of Radiation Oncology, Shandong Cancer Hospital and Institute, Shandong First Medical University and Shandong Academy of Medical Sciences, Jinan, China; ^2^ Department of Cardiology, Qilu Hospital Affiliated to Shandong University, Jinan, China

**Keywords:** cytokines, immune checkpoint inhibitors, predictive factors, adverse events, response

## Abstract

Recently, the overall survival (OS) and progression-free survival (PFS) of patients with advanced cancer has been significantly improved due to the application of immune checkpoint inhibitors (ICIs). Low response rate and high occurrence of immune-related adverse events (irAEs) make urgently need for ideal predictive biomarkers to identity efficient population and guide treatment strategies. Cytokines are small soluble proteins with a wide range of biological activity that are secreted by activated immune cells or tumor cells and act as a bridge between innate immunity, infection, inflammation and cancer. Cytokines can be detected in peripheral blood and suitable for dynamic detection. During the era of ICIs, many studies investigated the role of cytokines in prediction of the efficiency and toxicity of ICIs. Herein, we review the relevant studies on TNF-α, IFN-γ, IL-6, IL-8, TGF-β and other cytokines as biomarkers for predicting ICI-related reactions and adverse events, and explore the immunomodulatory mechanisms. Finally, the most important purpose of this review is to help identify predictors of ICI to screen patients who are most likely to benefit from immunotherapy.

## Introduction

Immune checkpoint inhibitors (ICIs) therapy has shown improved clinical responses and significant survival benefits in patients with locally advanced or advanced solid tumor types. Specifically, programmed cell death 1 (PD-1), programmed cell death ligand 1 (PD-L1), and monoclonal antibodies against cytotoxic T lymphocyte antigen 4 (CTLA-4) have been approved in the first-line and second-line treatment of various malignant neoplasms, including non-small cell lung cancer (NSCLC), melanoma, renal cell carcinoma (RCC), etc. (1–4). To date, there are six Food and Drug Administration (FDA)-approved ICIs for PD-1/PD-L1/CTLA-4, including pembrolizumab and nivolumab (anti-PD-1) and atezolizumab, durvalumab and avelumab (anti-PD-L1). However, the first-line objective response rate (ORR) of NSCLC treated by ICIs plus chemotherapy was approximately 48-58% (5, 6), and ICI monotherapy was only approximately 27-46% (7, 8). Thus, a large number of patients did not receive beneficial effects. Additionally, immune-related adverse events (irAEs), which occurs mostly in derma, digestive system, endocrine organs and lungs. The response rate of any grade of irAEs was 30% and ≥grade 3 was 5-10%, even 1% death rate (9, 10). Hence, biomarkers are pressing needed for identification patients who will benefit most from ICIs and avoid over treatment.

Predictive biomarkers for prognosis and adverse reactions of ICI treatment received more attention and have been widely explored in recent years. According to the simple being test, current biomarkers are mainly including tumor tissue biomarkers [e.g. PD-L1 expression (11, 12), tumor mutation burden (TMB) (13), MHC molecule expression (14, 15)], circulating immune cells biomarkers [e.g. CD4^+^T cells (16, 17), myeloid-derived suppressive cells (MDSCs) (18)] and soluble systemic immune/inflammatory biomarkers [e.g. lactate dehydrogenase (LDH) (19, 20), cytokines (21, 22)]. However, definite conclusions have not been reached. So far, PD-L1 expression based on assays on tumor cells is the only biomarker that is approved and extensively used for selecting patients for PD1/PD-L1 immunotherapy (23, 24). However, response was observed in PD-L1 negative patients and not all PD-L1 positive patients benefit (25). In addition, PD-L1 detection requires tissue samples obtained by an invasive means and local tissue may provide an incomplete insight of TME. Circulating biomarkers based on plasma offer an alternative non-invasive solution to address these weaknesses.

Cytokines (CK) are a series of low molecular weight soluble proteins, including interleukin (IL), interferon (IFN), tumor necrosis factor (TNF) superfamily, colony stimulating factors (CSF), chemokines, and growth factors (GF), secreted by immune cells (such as monocytes, macrophages, T cells, B cells, and NK cells) and some nonimmune cells (endothelial cells, epidermal cells, and fibroblasts) after induction by immunogens, mitogens, or other stimulants (26). Autocrine, paracrine, or endocrine cytokines bind to the receptors on the target cell membrane to trigger intracellular signals and change specific cellular functions (27, 28). Changes of cytokines levels regulate tumor microenvironment, change the proliferation and differentiation of immune cells, and even influence the metastasis of cancer cells (29, 30).

A series of studies have evaluated the level of baseline and changes of cytokines in various tumors patients treated with ICIs. Several preclinical model studies have shown that a combination of certain cytokine drugs and ICIs can improve the prognosis (31, 32). In this review, we retrospect the potential value of cytokines in predicting the efficacy and adverse reactions of immune checkpoint therapy (ICT) and intends to identify patients who will benefit from ICIs.

## TNF-α

Tumor necrosis factor (TNF) was named in 1975 by Carswell because TNF caused tumor bleeding and necrosis (33). TNF-α is mainly produced by monocytes and macrophages. Additionally, TNF-α is detected in tumor tissue and secreted by malignant tumor tissues or interstitial tumor cells. Soluble TNF-α (sTNF) and transmembrane TNF-α (tmTNF) are two forms of TNF-α. TNF-α binds to two receptors including TNFR1 and TNFR2. TNFR1 is ubiquitously expressed, and TNFR2 is expressed only in immune cells, neurons, and endothelial cells (34, 35). After binding to the receptors, TNF-α recruits death domain (DD) proteins, such as Fas-associated death domain (FADD) and TNFR associated DD (TRADD), activates apoptotic signal transduction pathways, and recruits TRAF family proteins, such as NF-kappa B and JNK, which accelerate cell proliferation and differentiation (36, 37). Moreover, TNF-α plays a key role in a number of systemic inflammatory diseases. Anti-TNF-α drugs are effective in the treatment of inflammation associated with a variety of autoimmune diseases, such as rheumatoid arthritis (RA), inflammatory bowel disease (IBD) (38), ankylosing spondylitis, and Behcet’s disease (39).

The associations of TNF-α with a response to ICIs was investigated in several studies. Tanaka et al. (40) showed serum levels of TNF-α were decreased in 6/9 malignant melanoma (MM) patients with complete remission (CR), partial remission (PR) or long-term stable disease (long SD), and elevated in six patients with progressive disease (PD) (P<0.05). It agrees with the preclinical study results that TNF induced resistance of immunotherapy (41). Several studies demonstrated the mechanism that TNF-α may acted as a negative biomarker. TNF-α upregulates the expression of PD-L1 in tumor cells and T cell immunoglobulin and mucin domain 3 (TIM-3) in CD8^+^ TIL (41, 42). TIM-3 has been reported as secondary immune checkpoints which limit the function of tumor reactive T cells (31, 43) and trigger TIL exhaustion (44). Additionally, TNF was shown to trigger CD8^+^ T cell activation-induced cell death (AICD) (45, 46). Blocking TNF and PD-1 increased the number and activity of tumor-infiltrating CD8^+^ TIL (31, 47) and enabled to achieve 75% survival *versus* 20% survival in the case of treatment with anti-PD-1 alone. In addition, Lim et al. found that TNF-α upregulates the expression of ubiquitin enzyme CSN5, which reduces the ubiquitination of PD-L1 and stabilizes its expression (41).

Different results have been reported in studies. Boutsikou et al. (21) reported increased TNF-α levels at the time before and 3 months after anti-PD-1 was correlated with improved response and prolonged survival (P=0.009) in 26 NSCLC patients treated with PD-1 inhibitors, while not association with PFS. Additionally, Ozawa et al. (45) showed no significant difference of TNF-α levels at time of before and 7 days anti-PD-1 in 10 NSCLC patients. However, no serious adverse effects were observed in patients with normal TNF-α levels. Baseline TNF-α may not act as an ideal biomarker for ICI treatment. Correlation between survival and non-synonymous TNF pathway mutations was not discovered in any cancer type (48). Changed TNF-α levels and suitable time for detecting plasma was needed further explored. A brief summary of clinical studies of the association of ICT response and irAEs with cytokines is respectively shown in **Tables 1**, **2**.

**Table 1:** Clinical studies of the association of ICT response with cytokines.

Cytokine	Authors	N=	Tumors	Immunotherapy	Results
TNF-α	Tanaka 2017 (40)	30	M	nivolumab	decreased levels of TNF-α associate with better reactivity
	Boutsikou 2018 (21)	26	NSCLC	pembrolizumab/nivolumab	increased TNF-α levels was correlate with improved response and prolonged survival
	Ozawa 2019 (45)	10	NSCLC	nivolumab/pembrolizumb	no relationship
IFN-γ	Yamazaki 2017 (49)	37	M	nivolumab	higher levels of IFN associate with improved response
	Boutsikou 2018 (21)	26	NCSLC	PD-1	increased levels of IFN-γ correlate with improved response and prolonged survival
	Hirashima 2019 (50)	29	NSCLC	PD-1/CTLA-4	low baseline IFN-γ level (<10 IU/ml)and decreased IFN-γ level associate with progression disease
	McNamara 2016 (51)	*	M	PD-1/PD-L1	ability of IFN-γ production by peripheral blood lymphocyte correlate with survival
	Costantini 2018 (52)	43	NSCLC	nivolumab	no correlation with baseline or variation IFN-γ levels
IL-6	Yamazaki 2017 (49)	35	M	nivolumab	higher IL-6 levels associate with improved response
	Hardy-Werbin 2019 (53)	84	SCLC	ipilimumab+chemotheray	baseline IL-6 levels lower than cut-off(3.65pg/ml) associate with higher OS
	Laino 2020 (54)	1296	M	nivolumab/ipilimumab	higher baseline IL-6 levels associate with shorter survival
	Ozawa 2019 (45)	10	NSCLC	nivolumab/pembrolizumb	elevated IL-6 or CRP associate with higher response rate
	Tsukamoto 2018 (55)	*	M	nivolumab	increased IL-6 levels associate with tumor progression
IL-8	Boutsikou 2018 (21)	26	NSCLC	nivolumab/pembrolizumab	increased levels of IL-8 correlate with prolonged OS
	Agullo-Ortuno 2020 (56)	27	NSCLC	nivolumab	increased levels of IL-8 associate with poor OS
	Sanmamed 2017 (57)	29	M	nivolumab/pembrolizumab	early increased IL-8 levels (2-4 weeks after anti-PD-1) associate with lower response rate
	Hardy-Werbin 2019 (53)	84	SCLC	chemotherapy plus ipilimumab	high level of IL-8 (≥13.82pg/mL) correlate with worse OS
	Yuen 2020 (58)	1445	UC, RCC	atezolizumab	high baseline levels of IL-8 correlate with poor efficiency
	Schalper 2020 (59)	1344	NSCLC, RCC	Nivolumab/nivolumab plus ipilimumab	high baseline levels of IL-8 associate with poor efficiency, cut-off (23pg/mL)
TGF-β	Feun 2019 (60)	24	HCC	Pembrolizumab	TGF-β≥200pg/mL assign as a poor indicator of response
	Mariathasan 2018 (61)	*	MUC	atezolizumab	increased TGF-β ligand1 (TGF-β1) and TGF-β receptor2 (TGF-βR2) levels correlate with poor response

TNF, tumor necrosis factor; IFN, interferon; IL, interleukin; TGF, transforming growth factor; M, melanoma; NSCLC, non-small cell lung cancer; UC, urothelial carcinoma; RCC, renal cell carcinoma; HCC, hepatocellular carcinoma; PD-1, programmed cell death protein 1; PD-L1, programmed cell death ligand 1; CTLA-4, cytotoxic T-lymphocyte antigen 4.

*mouse model.

**Table 2:** Clinical studies of the association of ICT irAEs with cytokines.

Cytokine	Authors	N=	Tumors	Immunotherapy	Results
IL-6	Tanaka 2017 (40)	30	M	Nivolumab	increased levels of IL-6 associate with psoriasiform dermatitis
	Okiyama 2017 (62)	20	M	Nivolumab/Pembrolizumab	increased levels of IL-6 associate with psoriasiform dermatitis
	Ozawa 2019 (45)	10	NSCLC	Ipilimumab	increased IL-6 level correlate with SAE rate
	Chaput 2017 (63)	26	M	ipilimumab	low baseline IL-6, IL-8, and sCD25 associate with colitis
	Valpione 2018 (64)	140	M	ipilimumab	low baseline IL-6 level act as independent predicted factor

IL, interleukin; M, melanoma; NSCLC, non-small cell lung cancer.

Preclinical studies targeted the combination of TNF and ICIs have received improved prognosis. For instance, compared to anti-PD-1 alone, the combination of anti-PD-1 and a TNF/TNFR1 gene defect or TNF blockade had better therapeutic benefit (75% survival *versus* <20% survival) in melanoma and lung cancer mouse models (31). Moreover, TNF inhibitor enalapril or anti-TNF monoclonal antibody decreased the risk of hepatitis and colitis induced by double checkpoint inhibition (44, 46). Anti-TNF antibodies, such as etanercept and infliximab, have been used in clinic to treat serious irAEs (65, 66).

## IFN-γ

Interferon-γ (IFN-γ) is a soluble cytokine dimer named for its ability to interfere with the growth of live viruses (67). IFN-γ is mainly produced by NK cells (68), activated T cells (69, 70), B cells (71, 72), and antigen-presenting cells [macrophages (73), monocytes (74), and dendritic cells (75)]. JAK/STAT is the main signaling pathway that mediates interferon-induced gene expression. IFN-γ binds to the cell surface receptors and triggers phosphorylation of JANUS family kinases JAK1 and JAK2. This phosphorylation activates signal transducer and transcriptional activator (STAT) protein. Response genes (e.g., IRF1) induced by STAT1 signaling enhance the transcription of the secondary response genes (76). IFN-γ is a multipotent cytokine with antiviral, proinflammatory, and immunomodulatory functions (77–79).

Recently, the role of IFN-γ in the prediction of the efficacy of immunotherapy was evaluated. A phase II clinical trial (49) of MM patients treated with nivolumab indicated that serum IFN levels in patients with responders (CR/PR were significantly higher than those in non-responders (PD) (P<0.0001). And a study reported increased levels of IFN-γ after 3 months of anti-PD-1 inhibitors treatment in 26 NSCLC patients was correlated with improved response and prolonged survival(P=0.002) (21). Another study of NSCLC patients who received ICIs showed low baseline IFN-γ level (<10 IU/ml)and decreased IFN-γ level after ICI treatment was associated with progression disease and immunotherapy-induced pneumonitis (50). Above all, increased levels of IFN-γ may acted as a positive biomarker for ICI therapy response. In fact, IFN-γ modulated the tumor micro-environment of ICI therapy (80). Damage of IFN-γ stimulus response is correlated with both primary and acquired resistance to ICI therapy (81, 82). In a mouse model, the ability of IFN-γ production by peripheral blood lymphocyte was significantly was a potent biomarker for survival of double PD-1/CTLA-4 blockade (51). However, Costantini et al. reported no correlation between baseline or variation IFN-γ level with 43 advanced NSCLC patients treated with nivolumab (52).

In addition, several clinical studies have shown that IFN-γ gene signature is related to prognosis. Brandon et al. showed that in advanced NSCLC, patients with four genetic IFN-γ^+^ signatures (interferon γ, CD274, LAG3, and CXCL9) had longer median OS (18.1-22.7m *vs* 6.5-7.7m) and six-fold higher objective remission rate (ORR) regardless of the immunohistochemical (IHC) PD-L1 status (83). In Keynote-012 clinical trial (84, 85), the average levels of six interferon-gamma-related genes (CXCL9, CXCL10, IDO1, IFNG, HLA-DRA, and STAT1) were associated with OS (p=0.0047) and PFS (p=0.0009) in patients with head and neck squamous cell carcinoma and gastric cancer treated with pembrolizumab. Moreover, the results of a phase II randomized controlled trial (86) showed high levels of the expression of IFN-γ-associated genes had longer survival in patients treated with atezolizumab. Thus, high expression of IFN-γ gene may predict a good prognosis of ICIs.

**Figure 1:**
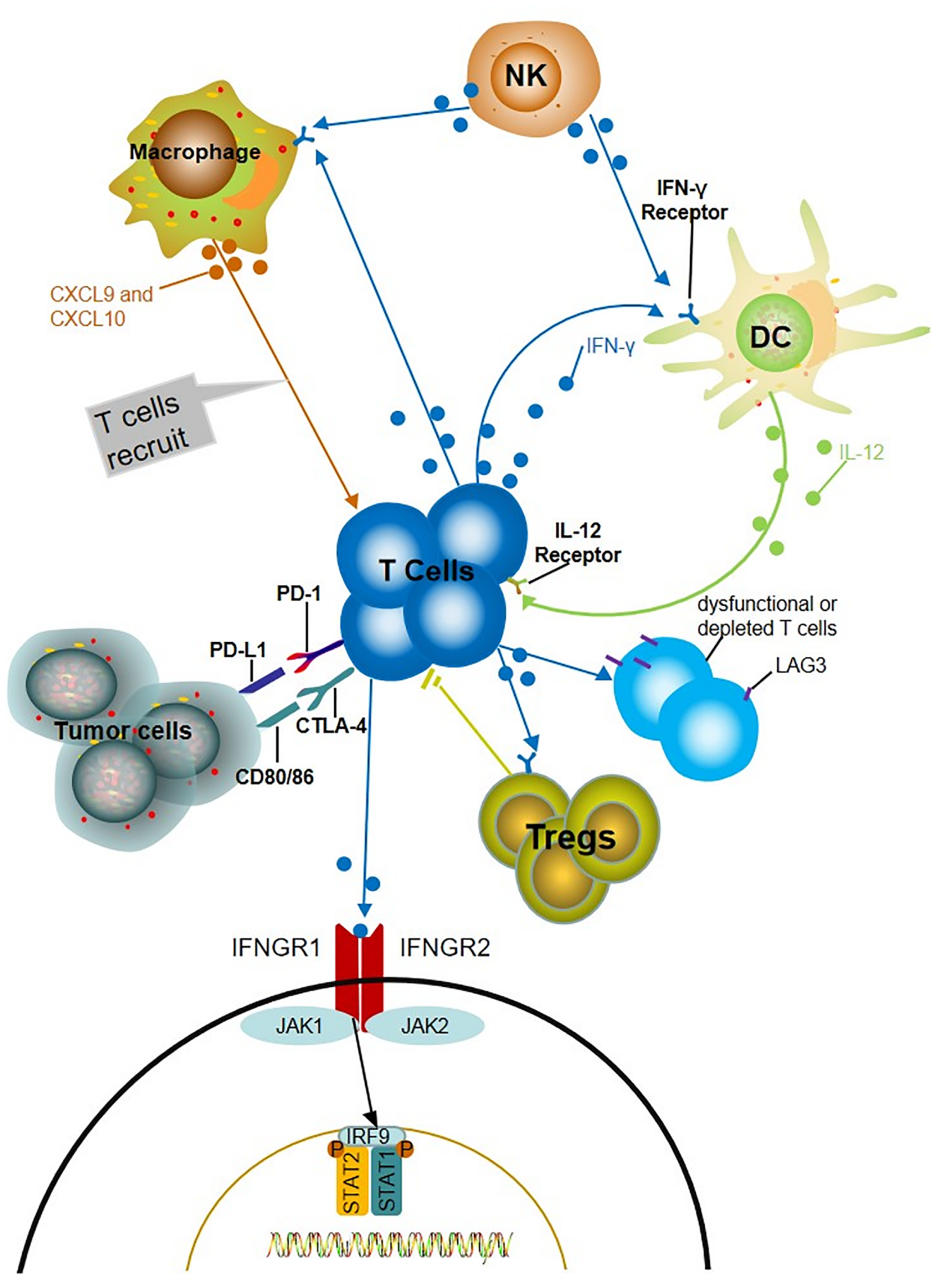
Potential mechanisms of IFN-γ predicting the prognosis of ICIs. The production of IFN-γ induces the increase of chemokine CXCL9 and CXCL10, which recruits more tumor reactive T cells and increased the level of IFN-γ. IFN-γ released by T cells stimulates neighboring dendritic cells (DCs) to produce IL-12, which in turn promotes the production of IFN-γ and forms a positive feedback loop. IFN- γ induces LAG3 which mainly express on dysfunctional or depleted T cells and induce immunosuppression. The fragility of Treg driven by IFN-γ produced by intratumoral Nrp1-/- treg limits the activity of CD4+Treg cells.

The potential mechanism of the relationship between IFN-γ and the response to ICIs has been demonstrated. ICIs promote the production of IFN-γ; then, IFN-γ increases tumor immunogenicity, inhibits tumor cell proliferation, and enhances the cytotoxic function of NK cells and CTL (87–90). Additionally, IFN-γ induces the secretion of CXCL9 and CXCL10 chemokines that recruit additional tumor-reactive T cells (83, 90). Moreover, IFN-γ released by T cells stimulates neighboring dendritic cells (DCs) to produce IL-12, which in turn promotes the production of IFN-γ and forms a positive feedback loop (91). Moreover, IFN-γ induces the expression of inhibitory receptor LAG3 (lymphocyte-activation gene 3), which mainly expressed on dysfunctional or depleted T cells and induces immunosuppression. Regulatory T cells (Tregs) are a functional subset of suppressor T cells that suppress anti-tumor immunity. IFN-γ produced by Nrp1-deficient (Nrp1^-/-^) Tregs decreases the stability of surrounding wild-type Tregs and enhances anti-tumor immunity (92) (**Figure 1**).

## IL-6

Interleukin-6 (IL-6) is produced by fibroblasts, monocytes/macrophages, T lymphocytes, B lymphocytes, epithelial cells, and a variety of tumor cells (93, 94). IL-6 is involved in cell survival, growth, immune regulation, and inflammation *via* the JAK/STAT signaling pathway (95). Moreover, IL-6 signaling plays a key role in carcinogenesis, inhibition of antitumor immunity, and promotion of tumor transmission in tumor environment (96–100). An increase in IL-6 is detected earlier than that in other cytokines and lasts for a long time during inflammatory reactions. Therefore, IL-6 can be used in early diagnosis of acute infection to evaluate the severity of an infection and prognosis. Drugs targeting IL-6, IL-6 receptors, or JAK have been approved by the FDA for the treatment of multicentric Casterman disease (siltuximab), arthritis (tocilizumab), and CART-induced CRS.

Several studies demonstrated associations between baseline IL-6 level and ICI response. Yamazaki et al. showed the IL-6 level pretreatment in responders (CR/PR) was remarkably higher than those in non-responders (PD) in 35 advanced MM patients treated with nivolumab (P=0.0007) (49). However, a study by Hardy-Werbin et al. reported the baseline IL-6 level lower than cut-off (3.65pg/ml) was significantly associated with higher OS compared with those with lower level (18.5m *vs* 9.5m) in patients treated with ipilimumab and chemotherapy (P=0.026) (53). And another study showed a similar result in which higher baseline IL-6 levels was associated with shorter survival (54). Overall, baseline IL-6 level was a strong prognostic marker of ICI treatment. In view of the limitations of small number patients and different types of ICI, the relationship between them needed further prospective clinical exploration. Furthermore, association between changes of IL-6 were also studied. Ozawa et al. (45) investigated the early changes in the cytokines (0-7 days before the treatment and after the treatment) with response in NSCLC patients treated with nivolumab. The response rate in patients with elevated IL-6 or CRP was 46%, which was significantly higher than that in patients with no increase (0%). A study by Tsuka-moto et al. reported that increased IL-6 levels were associated with tumor progression in melanoma patients treated with nivolumab (55). These findings suggest that increased level of IL-6 was a negative biomarker of prognosis with ICI treatment. Notably, combined anti-IL-6 and ICI treatment showed synergistic anti-tumor activity and improved prognosis, which helps to confirm the negative role of IL-6 in immunotherapy (55, 101, 102). And studies reported that IL-6 induced production of myeloid-derived suppressor cells (MDSCs) and resulted in immunosuppressive, which may explain the above phenomenon (103).

Associations of IL-6 with irAEs were also been studied extensively. Two studies reported increased level of IL-6 after nivolumab treatment was associated with psoriasiform dermatitis in patients with malanoma (40, 62). Tanaka et al. reported increased level of IL-6 was associated with higher incidence of psoriasis (p=0.018) in melanoma patients treated with nivolumab (40). SAEs rate in NSCLC patients treated with PD-1 inhibitors was 43% *vs* 0% in the group with increased IL-6 level compared to the normal IL-6 level group (45). Notably, a case report by Yoshino et al. showed decreased level of IL-6 and CRP accompanied with the clinical remission of colitis after corticosteroid treatment (104). In summary, the increased IL-6 level after ICIs treatment was an efficient biomarker for irAEs. Additionally, low baseline IL-6 level may use as a predictor for irAEs. Chaput et al. reported low baseline IL-6, IL-8, and sCD25 was associated with colitis related to ipilimumab treatment in melanoma patients (63). And another study by Valpione et al. showed low baseline IL-6 level act as independent predicted factor for irAEs (P=0.007) (64). In summary, high baseline level of IL-6 was a risk marker for irAEs, but the cut-off of high level needed further definition by more clinical trials. The elevation of IL-6 after ICI application may indicate the immune-sensitive individuals and play a predictive role before the occurrence of irAEs.

Interestingly, the association of irAEs and response was investigated by several studies (105–108). ORR in NSCLC patients with irAEs was reported higher than that in patients without irAEs (63.6% *versus* 7.4%, p <0.01) (109). Another study showed that patients with AE after nivolumab treatment had better ORR (37% *versus* 17%, p=0.17) and longer median PFS (6.4 *versus* 1.5 months, p=0.01) than those with no irAEs (108). The mechanism of associations of irAEs with response are unclear and need further investigation. Similar to cytokine release syndrome (CRS) after adoptive T cell therapy, ICIs may lead to hyper-physiological levels of proinflammatory cytokines, especially IL-6, resulting in irAEs.

## IL-8

Interleukin-8 (IL-8), also known as CXCL8, is a proinflammatory chemokine whose function was mediated by binding to two cell-surface G protein coupled receptors, termed CXCR1 and CXCR2 (110, 111). lL-8 was produced by immune cells (including macrophages, neutrophils and T cells) and non-immune cells (including epithelial and endothelial cells) (112, 113). IL-8 is identified as a neutrophil-activating cytokine and stimulated by hypoxia/anoxia, death receptors (Fas, DR5), and a variety of cellular stresses (114). Literatures reported IL-8 expression was higher in various tumor than healthy tissues (115). In addition, IL-8-CXCR1/2 pathway play an important role in tumor progression and metastasis (116), and activating proliferation of endothelial cells in tumor vasculature and induced vascular building was mainly mechanism (117).

Boutsikou et al. reported that increased level of IL-8 (3 months after immunotherapy) was correlated with prolonged OS (P=0.015) in 26 NSCLC patients treated with first or second line anti-PD-1 (nivolumab or pembrolizumab) (21). With controversial result, A study showed the increased level of IL-8 (2 months after immunotherapy) was associated with poor OS (P=0.025) (56). Sanmamed et al. analyzed early increased IL-8 level(2-4 weeks after anti-PD-1) was associated with lower response rate in patients with MM (P<0.001) and NSCLC (P=0.001) with high sensitivity and specificity (57). Significantly, IL-8 level maintains a level below the baseline when tumor with pseudo-progression, but progressively increased when tumor with a real progression. These findings consist with the conclusion that IL-8 level reflect the tumor burden (118).

The relationship between baseline IL-8 levels have also been discussed. Higher baseline levels of IL-8 were associated with poor OS in SCLC patients whatever treated with chemotherapy alone (n=47) or chemotherapy plus ipilimumab (n=37). In ipilimumab cohort, patients with high level of IL-8 (≥13.82pg/mL) had a worse median OS (5.3m *vs* 17m) (53). In the study by Yuen et al. demonstrated that high baseline levels of IL-8 were correlated with poor efficiency by evaluating 1445 patients in metastatic urothelial carcinoma (mUC) and metastatic renal cell carcinoma (mRCC) from three large atezolizumab trails (58). Similar results were acted by assessing 1344 patients (NSCLC and RCC) treated with nivolumab or nivolumab plus ipilimumab and determined cut-off (23pg/mL) may guide clinical application of ICIs (59). High baseline and early increased IL-8 levels may act as a strong negative biomarker for ICI therapy.

## TGF-β

Transforming growth factor-β (TGF-β) induces transformation and growth of certain fibroblasts in combination with epidermal growth factor (EGF) (119). In addition to TGF-β, the TGF-β superfamily includes activin, inhibin, bone morphogenetic protein (BMP), and growth and differentiation factor (GDF). Cells with active differentiation often contain high levels of TGF-β, including osteoblasts, kidney, bone marrow, and fetal liver hematopoietic cells. Activated T cells, B cells, and numerous types of tumor cells can secrete TGF-β. TGF-β receptors (TGF-βR), including types I, II, and III, are involved in signal transduction. Typical TGF-β signal transduction includes TGF-β binding to TGF-βRII, which recruits and phosphorylates TGF-βRI. Then, these receptors phosphorylate smad2, smad3, and smad4 to induce gene transcription and expression (120, 121). TGF-β signal can be transduced by the MAPK, PI3K, and Rho-GTP pathways (122, 123). The TGF-β pathway plays an important role in the regulation of cell proliferation, growth, differentiation, apoptosis, and autoimmunity (124). Due to the diversity of TGF-β functions, dysfunctions of the TGF-β signal transduction are associated with many diseases, including systemic sclerosis, fibrosis, hereditary diseases, and cancer (125–129).

TGF-β has been shown to be associated with ICI response. A prospective phase II study showed mean TGF-β levels was higher in non-responders than responders (1071.8 pg/mL *vs* 141.9 pg/mL) in 24 patients with advanced liver cancer treated with pembrolizumab. And TGF-β≥200pg/mL was assigned as a poor indicator of response with pembrolizumab (60). The median PFS in patients with TGF-β levels <200 pg/mL compared with TGF-β level ≥200 pg/mL was 2 months *versus* >25 months. Mariathasan et al. reported increased TGF-β ligand1 (TGF-β1) and TGF-β receptor2 (TGF-βR2) levels was correlated with non-response (P=0.00011) and OS (P=0.0096) in patients with metastatic urothelial cancer (MUC) treated with atezolizumab (61). To sum up, increased TGF-β level acted as a negative biomarker of response of ICIs treatment. In general, TGF-β plays an inhibitory role in normal cells and early cancer cells, including cell cycle arrest and apoptosis (122). However, in advanced cancer, TGF-β can be used as a cancer-promoting factor to enhance tumorigenesis, including immunosuppression, tumor metastasis, and drug resistance (128, 129). Several studies have shown that the abnormal TGF-β signaling pathway may affect immune regulation, including antigen presentation, differentiation of CD4 Th1 cells, infiltration and proliferation of CD8^+^ T cells, and production of long-term memory T cells (130–134). These effects may result in low response and poor prognosis of ICT.

A combination of anti-PD-L1 and TGF-β antibody was studied to determine the negative effects of TGF-β on ICT in several studies. In a mouse model of urothelial cancer (UC) (61), combined application of anti-PD-L1 and TGF-β antibody reduced TGF-β signal transduction in stromal cells and promoted T cell into the tumor. An increased number of infiltrating CD8^+^ T cells resulted in tumor suppression. In addition, ICI monotherapy produce unsatisfactory results in phase II and III trials (135, 136). However, Jiao et al. (137) demonstrated that anti-CTLA-4 combined with anti-TGF-β therapy enhances the response to ICI therapy in a bone metastasis model of CRPC. Similarly, ICI combined with TGF-β antibody resulted in improved prognosis compared to that in ICI monotherapy in the experiments (22, 138). Interestingly, Lan et al. (139) designed a novel bifunctional drug M7824 which composed of PD-L1 monoclonal antibody and the extracellular domain of human TGF-βRII. M7824 is anti-PD-L1 and promote the activation of CD8^+^T cells and NK cells resulting in a higher OS and PFS (139, 140).

## Other IL Family Members

The relationships between IL family members, including IL-1β, IL-2, IL-4 and IL-10, and ICT response were evaluated in a number of studies. Boutsikou et al. assayed cytokines by flow cytometry in patients with NSCLC receiving pembrolizumab or nivolumab before treatment and 3 months after the treatment. The results showed that an increase in the levels of IL-1β (p=0.038), IL-2 (p=0.011), IL-4 (p=0.018), IL-6 (p=0.014), IFN-γ (p=0.00), and TNF-α (p=0.006) was associated with increased response rate (RR) (21). In another study, 65 cytokines were profiled in patients with unresectable stage III or IV melanoma treated with anti-PD-1 monotherapy (cohort 1) or anti-PD-1 plus anti-CTLA-4 (cohort 2). The results showed that the expression levels of IL-2 (p=0.041) in cohort 1 and TNF-α (p=0.0189) in cohort 2 were associated with OS (22). Additionally, several studied validated that high baseline levels of IL-2 and IL-4 are correlated with higher OS (53, 57). Studies summary is in **Table 3**. Increased cytokines mentioned after ICT related to better response and OS. Generally elevated levels of cytokines may reveal extensive immune activity, therefore, predicting good prognosis. Moreover, depletion of TILs is one of the possibly reason for patients with ICIs ineffectiveness (142). A study reported that IL-10–Fc enhanced immunotherapies by accelerating oxidative phosphorylation (OXPHOS) to reactive T cells and directly expand terminally exhausted CD8+ TILs (143). And in microsatellite stable (MSS) colorectal cancer (CRC) mouse models, IL-17A blocking combined with ICIs showed improved efficiency (144).

**Table 3:** Clinical studies of the association of ICT response with other cytokines.

Authors	N=	Tumors	Immunotherapy	Cytokines
Boutsikou 2018 (21)	26	NSCLC	nivolumab/pembrolizumab	IL-1β, IL-2, IL-4, IL-6, IL-8, IFN-γ, TNF-α
Lim 2019 (141)	147	M	PD-1+CTLA-4	IL-2, IL-8, TNF-α
Hardy-Werbin 2019 (53)	84	SCLC	chemotherapy plus ipilimumab	IL-2, TNF-α, IL-8, IL-4

NSCLC, non-small-cell lung cancer; M, melanoma; SCLC, small-cell lung cancer; IL, interleukin; IFN, interferon; TNF, tumor necrosis factor; PD-1, programmed cell death protein 1; CTLA-4, cytotoxic T-lymphocyte antigen 4.

The associations of the levels of IL and irAEs were also investigated. Tarhini et al. demonstrated that elevated baseline IL-17 levels were significantly associated with high risk of grade 3 diarrhea or colitis (p=0.02) in patients with advanced melanoma receiving neoadjuvant ipilimumab therapy (145). In another study, increased 11 cytokines (G-CSF, GM-CSF, fractalkine, FGF-2, IFNα2, IL-12p70, IL-1a, IL-1β, IL-1RA, IL-2, and IL-13) were all associated with severe toxicity and they integrated a CYTOX (cytokine toxicity) score which with AUC 0.68 at PRE (95% CI, 0.51-0.84, p=0.037) and 0.70 at EDT (95% CI, 0.55-0.85, p=0.017) (141). Additionally, a study reported that decreased IL-10 level in patients after anti-CTLA-4 treatment was associated with pancreatitis and uveitis, but the level of IFN-γ was not associated with irAEs (146). **Table 4** lists these studies. To sum up, these studies demonstrated lower baseline and increased levels of extensively cytokines after ICI treatment were associated with irAEs.

**Table 4:** Clinical studies of the association of ICT irAEs with other cytokines.

Authors	N=	Tumors	Immunotherapy	Cytokines
Tarhini 2015 (145)	147	M	PD-1+CTLA-4	CYTOX score(G-CSF,GM-CSF,Fractalkine,FGF-2,IFNα2, IL-12p70, IL-1a, IL-1β, IL-1RA, IL-2, IL-13)
Lim 2019 (141)	65	no limit	PD-1/PD-L1/CTLA-4	CXCL9, CXCL10, CXCL11, CXCL19
Sun 2008 (146)	16	bladder cancer	CTLA-4	IL-10

M, melanoma; G-CSF, granulocyte colony-stimulating Factor; GM-CSF, granulocyte-macrophage colony-stimulating factor; FGF, fibroblast growth factor; IL, interleukin; CXCL, C-X-C motif chemokine ligand; PD-1, programmed cell death protein 1; PD-L1, programmed cell death ligand 1; CTLA-4, cytotoxic T-lymphocyte antigen 4.

## Conclusion

Low efficiency and high irAEs occurrence urgently require biomarkers to identify patients that benefit from the immune checkpoint inhibitors therapy. Cytokines produced by immune cells and tumor cells in the tumor microenvironment may acted as a suitable candidate biomarker due to its no-invasive obtaining, easy for dynamic monitoring and cost-effective. The investigations of TNF-α, IFN-γ, IL-6, IL-8, TGF-β and other cytokines as predictors of the responses and adverse events of ICT produced encouraging results. Increased level of IFN-γ and IFN-γ pathway genes always acted as positive biomarkers for response and irAEs, while high baseline and increased level of IL-8, increased level of IL-6 and TGF-β was negative biomarkers. TNF-αwas generally regarded as an negative biomarker, but its predictive function is needed further exploration. Negative cytokines in the tumor microenvironment induce immunosuppression and even adverse reactions in ICT by regulating T cells, B cells, and related immune checkpoints. Thus, the combinations of cytokine drugs and ICIs improved prognosis and resulted in a number of ongoing clinical trials. However, it is unlikely a single cytokine will be sufficient to predict immunotherapy response and irAEs for the complexity of TME and interaction between cytokines. In future, development of multifactorial synergistic predictive markers is necessary to achieve individualized treatment and minimize adverse reactions.

## Author Contributions

HZ designed the study. MW collected literatures together with XZ and SX and drafted the manuscript. JG and JL coordinated. All authors contributed to the article and approved the submitted version.

## Funding

This work was supported by Innovation Project of Shandong Academy of Medical Sciences (2019-04) and the Academic Promotion Program of Shandong First Medical University [grant number: 2019ZL002]; the National Natural Science Foundation of China [grant number: 81972862]; and CSCO-Pilot Cancer Research Fund [grant number: Y-2019AZZD-0352]; and Key Research and Development Program of Shandong Province: Study on the role and mechanism of myocardial -AR in cardiac radiation injury [grant number: 2018GSF118067].

## Conflict of Interest

The authors declare that the research was conducted in the absence of any commercial or financial relationships that could be construed as a potential conflict of interest.
